# Long-term benefit of immunotherapy in a patient with squamous lung cancer exhibiting mismatch repair deficient/high microsatellite instability/high tumor mutational burden: A case report and literature review

**DOI:** 10.3389/fimmu.2022.1088683

**Published:** 2023-01-10

**Authors:** Na Li, Zixuan Wan, Dongyan Lu, Ruilian Chen, Xiaowei Ye

**Affiliations:** ^1^ First Clinical Medical College, Guangzhou University of Traditional Chinese, Guangzhou, China; ^2^ Department of Oncology, The First Affiliated Hospital of Guangzhou University of Traditional Chinese Medicine, Guangzhou, China

**Keywords:** mismatch repair deficiency, high microsatellite instability (MSI-H), high tumor mutational burden, squamous lung cancer, immunotherapy

## Abstract

Genetic mutations that render mismatch repair defective may result in microsatellite instability, which is common in colorectal carcinomas and gastric cancers as well as Lynch syndrome. Mismatch repair deficiency/high microsatellite instability (dMMR/MSI-H) predicts the tumor response to immune checkpoint inhibitors. However, few studies have evaluated the efficacy of immune checkpoint inhibitors in non-small cell lung cancer (NSCLC) patients with dMMR/MSI-H. In this work, we present a patient with advanced squamous lung cancer with dMMR/MSI-H and a high tumor mutational burden (TMB-H) who obtained a long-term benefit from immunotherapy. NSCLC patients with dMMR/MSI-H/TMB-H may thus benefit from immune checkpoint inhibitors.

## Introduction

Lung cancer is one of the most common cancers worldwide and has the highest mortality of all cancers (1). Immunotherapy has effectively treated many solid tumors. Programmed cell death-protein 1 (PD-1)/programmed death-ligand 1 (PD-L1) inhibitors have increased the 5-year survival rate for non-small cell lung cancer (NSCLC) patients by 20-30%. However, it remains challenging to find patients exhibiting long-term responses to immunotherapy in the present era of precision medicine. The predictive biomarkers of immune checkpoint inhibitor efficacy include the tumor cell surface PD-L1 expression level, tumor mutational burden (TMB), mismatch repair deficiency/high microsatellite instability (dMMR/MSI-H), and tumor-infiltrating lymphocytes (2–4).

Defects in DNA mismatch repair may lead to microsatellite instability, and subsequently a high TMB (TMB-H) and PD-L1 overexpression. The presence of dMMR/MSI-H was first detected in patients newly diagnosed with colorectal cancer. dMMR/MSI-H was closely associated with the immunotherapy response of solid tumors, particularly colorectal cancer, gynecological cancers, and gastric cancer (5–7). Accordingly, pembrolizumab and nivolumab were approved for the treatment of solid tumors with dMMR/MSI-H by the United States Food and Drug Administration (FDA) in 2017 (8, 9). Furthermore, pembrolizumab was approved as the first-line treatment of unresectable or metastasized colorectal cancer with dMMR/MSI-H in 2020 (10). However, dMMR and MSI-H are rare in patients with NSCLC. Little evidence bears on the question of whether dMMR/MSI-H/TMB-H predicts the effects of immune checkpoint inhibitors on lung cancer. In addition, the variability of microsatellite instability tests in terms of both the biologicals employed and the techniques renders it difficult to acquire usable data. Immunohistochemistry testing of MMR may yield inconsistent results for a particular germline mutation, recent studies have proposed that this may be attributable to somatic mutations (11). Next-generation sequencing (NGS) technology analyzes vast numbers of loci, simultaneously identifying tumor mutations and MSI. According to the European Society for Medical Oncology Congress (ECMO) recommendation, NGS is suggested to test MSI for rare cancer patients not belonging to the spectrum of Lynch syndrome (12). Here, we report a case of local advanced dMMR/MSI-H/TMB-H squamous cell lung cancer with a durable response to toripalimab treatment.

## Case presentation

**Figure 1:**
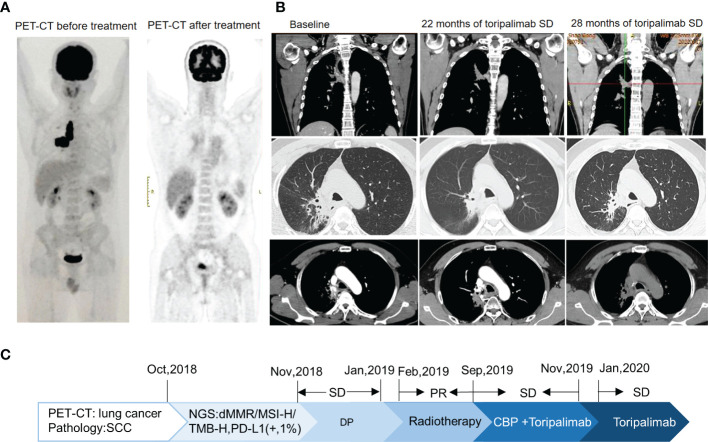
Imaging changes. **(A)** For PET-CT at diagnosis versus PET-CT after comprehensive treatment, the overall efficacy was PR. **(B)** CT images of Toripalimab during the different treatment periods. **(C)** The timeline of the main events of this case report.

**Figure 2:**
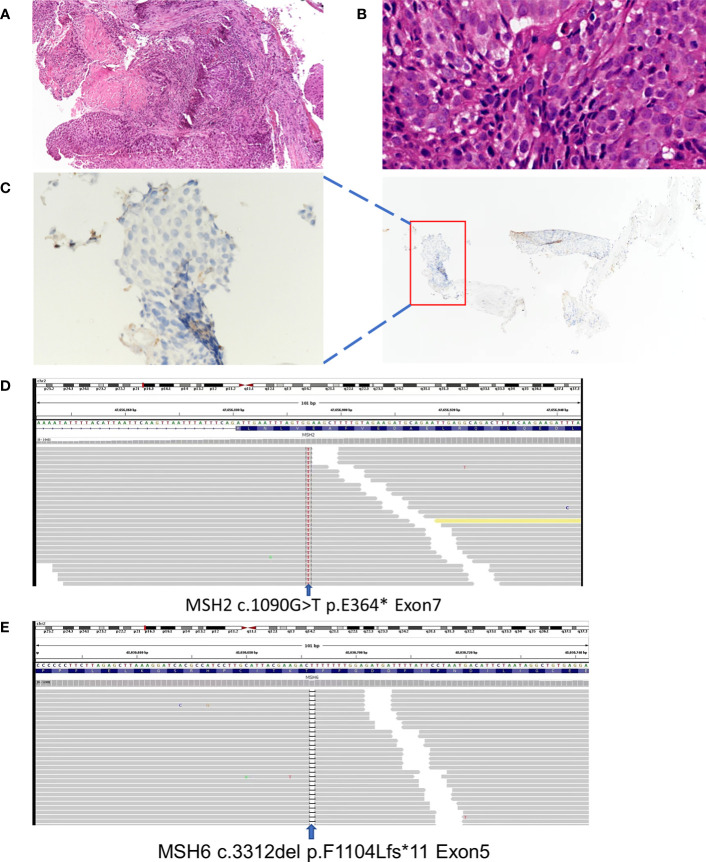
Basic information: **(A)** Lung tumor tissue stained with hematoxylin and HE, (magnification, ×100) **(B)** Lung tumor tissue stained with hematoxylin and HE, (magnification, ×400) **(C)** PD-L1 immunohistochemical (IHC) pathology, (magnification, ×400). **(D)** Next-generation gene sequencing revealed mutations in theMSH-2. **(E)** Next-generation gene sequencing revealed mutation in the MSH-6.

A 53-year-old non-smoker presented with an irritating cough, chest tightness, and shortness of breath for 5 months. There was no family history of tumors. Chest computed tomography (CT) showed a lesion near the right upper hilum with right upper pulmonary bronchial occlusion, and multiple lymph node metastases in the right hilum and mediastinum. Positron emission tomography-CT (PET-CT) showed that the lesion involved the adjacent pleura and bronchi of the right upper lobe. No other metastases were detected (**Figure 1A**). The patient underwent fiber optic bronchoscopy and biopsy of the tumor. Pathological examination of the tissue from the right upper lung revealed moderately differentiated squamous cell carcinoma (**Figures 2A, B**). The patient was classified with T3N2M0 lung cancer (stage IIIb) by reference to edition 8 of the Union for International Cancer Control (UICC). The VENTANA SP263 immunohistochemistry assay for PD-L1 expression revealed a tumor proportion score of 1% (**Figure 2C**). Paraffin-embedded tumor tissues with 60% of tumor purity and matched blood were subjected to genomic profiling using an NGS panel of 437 cancer-related genes (3D Medicines Inc, Shanghai, China). Somatic mutations in MSH2 (p.E364*Exon7) (**Figure 2D**), MSH6 (p.F1104Lfs*11Exon5) (**Figure 2E**), TP53 (p.R213*Exon6), and PTEN (p.R130QExon5) were found (**Table 1**). Further, NGS. A small panel next generation sequencing (SPANOM) algorithm covering 100 microsatellites is used for MSI determination (13). The SPANOM algorithm calculates the percentage of abnormal fragments of many bit sequences. According to the international standard of MSI testing (14), MSI-H greater than 0.4 is considered as MSI-H, otherwise it is considered as MSS. NGS revealed high MSI and high TMB (29.84 muts/Mb). The genetic tests thus revealed concurrent dMMR, MSI-H and TMB-H. The patient achieved stable disease (SD) after four courses of docetaxel plus cisplatin chemotherapy and radiotherapy. The patient received the first cycle of the immunotherapeutic toripalimab in October 2019. In March 2022, PET-CT showed an inactive or substantially suppressed tumor in the right upper lobe near the lung hilum (**Figure 1A**). At the time of writing this report (August 2022), the patient has been followed for 33 months and continues to evidence a durable response with toripalimab treatment (**Figures 1B, C**).

**Table 1:** Somatic testing results.

Mutation type	Gene	cHGVS	pHGVS	VAF(%)	Copy number
Somatic	MSH6	c.3312del	p.F1104Lfs*11Exon5	7.85	
	MSH2	c.1090G>T	p.E364* Exon7	17.11	
	PTEN	c.389G>A	p.R130Q Exon5	17.12	
	FAT1	c.5845G>T	p.E1949* Exon10	12.03	
	TP53	c.637C>T	p.R213* Exon6	12.67	
	NOTCH3	c.5260C>T	p.H1754Y	12.13	
	NTRK2	c.750A>T	p.L250F	14.31	
	KMT2A	c.2318del	p.P773Rfs*8	8.01	
	NTRK3	c.1906G>A	p.A636T	98	
	IRS2	c.998C>A	p.P333H	6.75	
	TYK2	c.1264G>A	p.G422S	10.53	
	PRDM1	c.1751T>C	p.F584S	9.04	
	SMARCA2	c.3786G>T	p.R1262S	12.34	
	FLT1	c.376A>G	p.I126V	7.38	
	TBX3	c.459G>T	p.M153I	11.83	
	TBX3	c.1874G>A	p.R625H	8.45	
	GLI3	c.318dup	p.T107Dfs*32	7.19	
	ETV6	c.496G>C	p.V166L	8.77	
	SMARCA4	c.708_713del	p.G243_P244del	6.20	
	CCND1	c.545C>T	p.A182V	4.52	
	ZNF703	c.150C>G	p.I50M	13.82	
	ZNF217	c.507G>A	p.W169*	10.22	
	PMS2	c.1667A>C	p.E556A	10.64	
	EP300	c.4228A>G	p.R1410G	8.21	
	BCOR	c.573G>T	p.W191C	16.58	
	CIC	c.4594G>A	p.E1532K	11.10	
CNV	CCND1	Copy number gains		N/A	7
	FGF19	Copy number gains		N/A	5
	FGF3	Copy number gains		N/A	5
	FGF4	Copy number gains		N/A	5
Fusion	ARID1A	exon12-18 del 1001bp		N/A	
	LRP1B	exon30-32 del 420bp		N/A	
MSI-H					
TMB-H(29.84 Muts/Mb)					
PD-L1 (+, 1%)					

VAF, Variant alle frequency.

## Discussion

The MMR system plays an important role in preserving genomic integrity by identifying and repairing base mismatches and deletions in the DNA damage response signal network and maintaining the structural integrity and stability of DNA (15). An MMR deficiency can be caused by somatic or germline mutations in MMR genes, including MLH1, MSH2, MSH6, and PMS2. The mutational frequencies of MSH2 and MSH6 in lung cancer are 1.4% and 1.6%, respectively, and 0.8% and 1.4%, respectively, in lung squamous cell carcinoma. An MMR deficiency has been considered to be the second most common cause of hereditary ovarian cancer after HRD (16). Recent studies have shown that high levels of MSH6, MLH1, and PMS2 prolong overall survival after ovarian cancer, and the status of the MMR gene has prognostic significance for those receiving platinum chemotherapy (17). Pathogenic germline mutations in MMR system genes occur mainly in Lynch syndrome-associated tumors, usually colorectal cancers (18), endometrial carcinomas (19), and gastric cancers (20). However, mutations in MMR genes were found in only 5% of lung cancer patients (21). Deficient MMR leads to mismatch accumulation during DNA base replication and consequently MSI-H.

**Figure 3:**
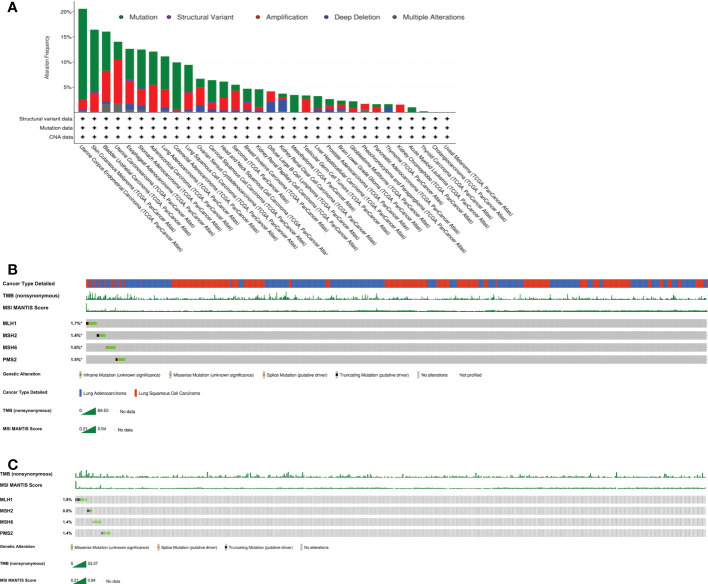
Characteristics of MLH1,MSH2,MSH6 and PMS2 mutations from cBioPortal. The data is analyzed by https://www.cbioportal.org in all cancer types **(A)**, the lung cancer cohorts **(B)** and the squamous lung cancer cohorts **(C)**.

A microsatellite is a tandemly repeated genomic DNA sequence. Microsatellite instability refers to the appearance of new microsatellite alleles at a certain tumor microsatellite locus *via* the insertion or deletion of repetitive units. Microsatellite instability produces many frameshift mutations, changing the downstream codons thus the encoded peptides. These new antigens are highly immunogenic and trigger the activity of tumor-infiltrating lymphocytes. Sensitivity to immune checkpoint inhibitors (ICIs) rises as a result of such modifications (22). As a true of dMMR, MSI-H is most common in colorectal cancers (23) and endometrial cancers (24), and is rare in lung cancers. DeMarchiP et al. (25) showed that the morbidity rate of MSI-H lung cancer was only 0.8% (4 cases) among 480 cases of lung adenocarcinoma, and there was only one mismatch repair defect in 4 cases of lung cancer evidencing high-level instability. Furthermore, there was only 1 case of MSI-H (0.19%) among 524 cases of NSCLC (25). Another study showed that the prevalence of MSI-H was 0.53-1.0% and 0.60% in lung adenocarcinomas and lung squamous cell cancers respectively (21). An analysis of the cBioPortal database revealed significant variations in the frequencies of MLH1, MSH2, MSH6, and PMS2 mutational changes among various tumor types, the figure was about 21% for endometrial carcinoma, 9% for lung squamous cell carcinoma, and 11% for lung adenocarcinoma (**Figure 3A**). We showed the genomic characteristics of MLH1, MSH2, MSH6, and PMS2 mutations in the lung cancer cohort and the lung squamous cell carcinoma cohort (**Figures 3B, C**). Here, we reported a rare patient with squamous cell lung cancer with dMMR/MSI-H/TMB-H who maintained a long-term response to toripalimab treatment. Therefore, dMMR/MSI-H NSCLC may benefit from immunotherapy, and dMMR/MSI-H status may serve as predictor of the response to immunotherapy for NSCLC patients.

dMMR/MSI-H cancers are characterized by a strong mutator phenotype, high somatic mutation load, lymphocyte infiltration, and high expression of immune checkpoint markers (26). These cancers often produce new antigens to promote immune cell infiltration and upregulation of immune checkpoint molecules to promote tumor immune escape. PD-1 receptor inhibitors are used for immunotherapy and antagonize the aforementioned effects. The production of new antigens by the MSI tumor underlies their increased immune activity. Because of the enhanced anti-tumor efficiency, patients with MSI tumors show a better prognosis and higher survival rate compared with non-MSI tumors. Gatalica et al. (27) found that 22% of patients with cancer of unknown primary (CUP) exhibited at least 5% PD-L1 expression; in the same study, 1.8% of patients exhibited high MSI and 11.8% had a high TMB (≥17 muts/Mb). Generally, CUP patients with a TMB >10 muts/Mb tend to show better outcomes when treated with ICIs (28).

Several large-scale clinical studies have demonstrated a role of dMMR/MSI-H in immunotherapy. In the KEYNOTE-177 study, patients with dMMR/MSI-H colorectal cancer were randomized to treatment with pembrolizumab or chemotherapy (29). The median survival times in the chemotherapy and pembrolizumab groups were 8.2 and 16.5 months, respectively. In the KEYNOTE-016 study, the objective response rate in the pembrolizumab group reached 36% in dMMR/MSI-H colorectal cancer and 46% in other cancers. Based on these remarkable results, pembrolizumab was approved by the FDA for the treatment of unresectable or metastasized cancers with dMMR/MSI-H (30). This is the first time that a predictive biomarker has been used to define an indication irrespective of the primary tumor site. In the CheckMate-142 study (9), the objective response rates of a nivolumab group and a nivolumab plus ipilimumab group were 34% and 58% for dMMR/MSI-H colorectal cancer patients, respectively. Furthermore, the nivolumab plus ipilimumab group had a disease control rate of 81%, 12-month progression-free survival (PFS) rate of 71%, and 12-month overall survival rate of 85% (31). As a result, nivolumab was approved by the FDA for the treatment of dMMR or MSI-H metastatic colorectal cancer patients aged ≥ 12 years who exhibit disease progression after treatment with fluorouracil, oxaliplatin, and irinotecan. MMR status in thus considered to be an important predictive biomarker of the response in patients with malignant tumors, regardless of the cancer type.

Few studies have evaluated the relationship between MMR gene mutations and immunotherapy response in NSCLC patients. Mendez (32) revealed that the median PFS of NSCLC patients with a loss of MMR expression was 8.0 months, which was longer than in those with retained MMR expression. We present a squamous lung cancer patient with somatic mutations in MSH2 and MSH6 who obtained a durable clinical benefit with toripalimab treatment.

Anti-PD-1/PD-L1 immunotherapies have improved the survival of patients with advanced lung cancer (33). PD-L1 is considered to be a predictive biomarker in clinical practice. However, not all individuals evidencing high-level PD-L1 expression obtain clinical benefits from immunotherapy (34). Our patient still obtained a long-term benefit from toripalimab although the PD-L1 expression level was only 1%. Thus, the use of PD-L1 as a predict biomarker still has several limitations; other potential markers are required.

Researchers continue to be intrigued by the susceptibility of TMB to immune checkpoint inhibitors; this topic is still being studied. A substantial statistical correlation between TMB and smoking habits has been described (35). Although our patient was a non-smoker, he had been exposed to passive smoke for more than 10 years, consistent with the statistical findings. Previous studies have shown that the TMB is associated with a good response to immune checkpoint inhibitors (36). This may be due to changes in the microsatellite sequence of patients with dMMR and a large number of tumor cell mutations, resulting in TMB-H and consequently the production of new antigens and lymphocyte mobilization. As a result, tumor growth and tumor lymphocyte infiltration, may be inhibited, leading to a good effect of immunotherapy (37). On the other hand, the immune system may be stimulated if ICIs are used to block immune checkpoint proteins. The more antigens a cell can be produce, the more the mutation; new immunogenic antigens on the surfaces of cancer cells enable T cells to recognize and eradicate such cells (4). Huang et al. (38) found that the median PFS was longer in patients with TMB-H lung cancer versus low TMB lung cancer (10.6 months vs. 3.9 months). This suggests that TMB is a potential biomarker of the response to the PD-1 and PD-L1 inhibitors in advanced NSCLC. Additionally, Rizvi et al. (39) revealed that a higher non-synonymous TMB in NSCLC is associated with a good long-term response to pembrolizumab treatment. The FDA approved pembrolizumab for the treatment of solid tumors with TMB≥10 muts/Mb. Although clinical evidence suggests that a higher TMB load indicates better immunotherapy efficacy, use of the TMB alone as a predictive biomarker has certain limitations (4).

In addition to the markers introduced above, other possible biomarkers have attracted interest in terms of their prognostic and predictive utility. A high level of tumor infiltrating lymphocytes (TILs) improves NSCLC survival (40). The predictive utility of TIL as a biomarker of immunotherapy has been confirmed. Ricci et al. (41) proposed that DNA damage repair (DDR) alterations could serve as potential predictive biomarkers in patients with solid tumors, including NSCLC. The human microbiota can also modify immunotherapeutic efficacy (42). Proton pump inhibitors and histamine-2-receptor antagonists can affect the immunotherapeutic efficacy in lung cancer by altering the gut microbiome (43, 44).

The FDA-approved immune checkpoint inhibitors for NSCLC treatment include pembrolizumab, nivolumab, and atezolizumab. In the KEYNOTE-024 study, pembrolizumab was used for untreated NSCLC without EGFR/ALK alterations and a PD-L1 tumor proportion score of 50% or greater. The pembrolizumab group had a two-fold greater 5-year survival rate compared with the chemotherapy group (31.9% vs 16.3%) (45). In the CheckMate078 trial, nivolumab treatment had greater efficacy and fewer side effects compared with docetaxel treatment for stage IIIB/IV NSCLC that progressed after platinum-containing dual-drug treatment (46). According to the analysis of PACIFIC trial, patients with unresectable stage III NSCLC who had received chemoradiotherapy showed a better survival advantage for durvalumab. Compared with the placebo group, the PFS of the durvalumab group was 16.8 months (47). The safeties of immune-based combinations, including nivolumab plus ipilimumab and pembrolizumab plus axitinib have also been studied (48). A growing number of immune medicines, including toripalimab, have been developed for the treatment of NSCLC.

In the CHOICE-01 study, the median PFS of the toripalimab plus chemotherapy group was superior to that of the chemotherapy group for locally advanced or metastatic NSCLC patients who were EGFR- and ALK-negative (8.4 vs. 5.6 months) (49). Furthermore, in the PD-L1 positive and negative subgroups, the risk of disease progression or death was decreased by 48% and 51%, respectively. The TMB-H (≥ 10muts/Mb) subgroup showed a better response to combination chemotherapy containing toripalimab and a lower risk of progressive disease or mortality compared with the TMB-L group (median PFS: 13.1 vs.5.5 months, HR=0.34). Our patient treated with toripalimab maintained SD over the entire 33-month follow-up period.

Concurrent dMMR/MSI-H and a TMB-H are rarely reported. We reviewed the literature and found several cases of MSI-H/TMB-H lung cancer patients treated with ICIs (**Table 2**). Kawashima et al. (50), Masuzawa et al. (51), and Vauchier et al. (52) reported that patients with MMR mutations and MSI-H achieved durable benefits from ICIs. In Han et al. (53), a lung cancer patient of dMMR/MSI-H/TMB-H status was treated with nivolumab for 3 months and obtained 19 months of PFS. In Long et al. (54), a lung cancer patient with an MSH6 mutation but a low TMB developed disease progression after receiving pembrolizumab for 6 months. However, all of the patients reported above had germline mutations in the MMR gene, thus both NSCLC and Lynch syndrome. Somatic mutation of the MMR gene accompanied by MSI-H/TMB-H remains rare in NSCLC patients.

**Table 2:** Clinical information and efficacy of ICIs in lung cancer patients with MSI-H/TMB-H.

Author, year	Histology Type	Age onset, y/Gender	MSI	MMR	TMB (Muts/Mb)	Immunotherapy/time	Follow-up	RECIST	PFS
Kawashima Y et al.2018	ADC	68/M	MSI-H	dMMR	ND	Nivolumab/ 4 cycles	22 months	PR	/
Masuzawa K et al., 2019	ADC	36/M	MSI-H	MLH1 (c.2180_ 2181del, p.His727Hisfs*5)	ND	Nivolumab/ 15 cycles	35 months	PR	/
Vauchier C et al., 2021	NOS	78/M	MSI-H	dMMR	45	Pembrolizumab/ 17 cycles	13 months	PR	/
Han Q et al., 2021	ADC	62/M	MSI-H	PMS2(c.1144+1G>A)	13.62	Nivolumab/ 4 cycles	19 months	PD	19 months
Long Y et al., 2020	SCC	76/M	ND	MSH6 (c.2552_2553dupGC, p.K852Afs*17)	8	Pembrolizumab/ 8 cycles	6 months	PD	6months

SCC, squamous; ADC, adenocarcinoma; NOS, non-small-cell carcinoma not otherwise specified; M, male; F, female; TMB, tumor mutational burden; MSI, microsatellite instability; MMR, microsatellite instability; Ms, Months; RECIST, response evaluation criteria in solid tumors; PR, partial response; PD, progressive disease; ND, not detected.

## Conclusion

A squamous cell lung cancer patient with a rare dMMR/MSI-H/TMB-H presentation evidenced a durable response to toripalimab treatment. He received long-term treatment with toripalimab after four cycles of chemotherapy and radiotherapy. ICIs may be optional treatment strategies in NSCLC patients with dMMR/MSI-H or TMB-H. Additionally, dMMR/MSI-H may serve as a biomarker predicting the response to ICIs by lung cancer patients. Further work is needed to investigate the efficacy of immunotherapy in squamous cell lung cancer patients with dMMR/MSI-H/TMB-H.

## Data availability statement

The original contributions presented in the study are included in the article/supplementary materials. Further inquiries can be directed to the corresponding authors.

## Ethics statement

Written informed consent was obtained from the individual(s) for the publication of any potentially identifiable images or data included in this article.

## Author contributions

Concept and design: NL, RC. Acquisition, analysis, and interpretation of data: NL, RC, ZW. Drafting of the manuscript: NL. Critical revision of the manuscript for important intellectual content: RC, DL, XY. Study supervision: XY. All authors contributed to the article and approved the submitted version.
